# Utilization of healthcare by immigrants in Canada: a cross-sectional analysis of the Canadian Community Health Survey

**DOI:** 10.1186/s12875-022-01682-2

**Published:** 2022-04-06

**Authors:** Nisanthini Ravichandiran, Maria Mathews, Bridget L. Ryan

**Affiliations:** 1 Department of Family Medicine, Schulich School of Medicine & Dentistry, University of Western Ontario, 1151 Richmond Street, London, ON N6A 5C1 Canada; 2 Department of Family and Community Medicine, University of Toronto, 500 University Ave, Toronto, ON M5G 1V7 Canada; 3 Department of Epidemiology and Biostatistics, Schulich School of Medicine & Dentistry, University of Western Ontario, 1465 Richmond Street, London, ON N6G 2M1 Canada

**Keywords:** Health care utilization, Health care access, Immigration, Primary care, Cross-sectional, Secondary data analysis, Regular doctor, Consultations, CCHS

## Abstract

**Background:**

Immigrants to Canada face unique barriers to health care, which leads to inequities in health care utilization. Lower utilization of health care by immigrants to Canada is associated with the deteriorating health of individual immigrants as well as increased costs to the health care system. The existing literature suggests that time since immigration is an important predictor for utilization of health care for Canadian immigrants; however, few studies have included this variable in their analysis. This study aims to examine the relationships between having a regular health care provider and time since immigration, and number of medical consultations in the past year and time since immigration.

**Methods:**

A secondary cross-sectional data analysis using Andersen and Newman’s Framework of Health Service Utilization and data from the 2015–2016 Canadian Community Health Survey (CCHS) was conducted to examine health care utilization among immigrants in Canada. We used multiple logistic regression to examine the relationship between time since immigration and having a regular physician and negative binomial regression to compare the number of consultations of recent (less than 10 years since immigration) and established (10 or more years since immigration) immigrants.

**Results:**

Eighty four percent of immigrant respondents to CCHS 2015–2016 had a regular health care provider. After controlling for other independent variables, established immigrants were 1.75 (95% confidence interval: 1.45–2.10) times more likely to have a regular health care provider compared to recent immigrants. Immigrants had a mean of 3.37 (standard deviation 4.53) medical consultations in the preceding year. There was no difference in the mean number of medical consultations by recent and established immigrants.

**Conclusions:**

After controlling for other independent variables, this study found that time since immigration had a significant effect on having a regular provider but not on number of consultations. Differences in health care utilization for recent and for established immigrants observed in this study may be partially explained by Canada’s evolving immigration policy and the economic and social integration of immigrants over time.

## Background

Despite the existence of publicly funded health insurance in Canada, numerous barriers exist to utilizing health care, especially for immigrants to Canada who represent a vulnerable segment of the population [1–3]. Immigrants to Canada who face barriers to utilization of health care have worse self-reported health status than immigrants who do not report barriers [2].

Having a regular doctor was the most frequently examined outcome measure among studies of health care utilization by immigrants. However, only a few studies examined the factors associated with having a regular doctor for immigrants to Canada. In a study based on data from the Canadian Community Health Survey (CCHS) from 2000 to 2010, amongst immigrants overall, white immigrants, women, those married/common-law/partner, those with less than college education, and those with higher income were more likely to have a regular doctor [4]. Another study based on the 2007–2008 dataset of the CCHS found that language proficiency was not associated with having a regular doctor for immigrants to Canada [5]. In a separate analysis of the 2002–2003 Joint Canada-United States Survey of Health, white immigrants were less likely to have a regular doctor than native-born white individuals [6]. Few studies have examined the number of visits to a health care provider by immigrants to Canada and only one study included number of medical consultations in the past year as an outcome variable [7]. This cross-sectional study [7] found that immigrants had more primary care visits in the past year relative to those who were Canadian-born. However, this was a small practice-based study in Ontario, and therefore may not be generalizable to the broader Canadian immigrant population.

The existing literature suggests that time since immigration is an important predictor for utilization of health care for Canadian immigrants [4, 6, 8, 9], with established immigrants being more likely to have a regular doctor than recent immigrants. However, many studies of utilization of health care by immigrants in Canada did not include time since immigration in the analysis [6, 7, 10–12].

The objectives of this study were to use recent data from the CCHS: 1) to examine the relationship between having a regular health care provider and time since immigration; and 2) to examine the relationship between number of medical consultations in the past year and time since immigration. Barriers to utilization of health care have real consequences in terms of both the health of immigrants and greater long-term costs to the health care system. Research on utilization of health care by immigrants to Canada is needed to help inform policy-making and optimize access and health outcomes.

## Methods

### Design

A secondary cross-sectional data analysis was conducted using the 2015–2016 dataset for the CCHS [13].

### Data source

This study used the public use microdata file (PUMF) from the 2015–2016 CCHS, which was retrieved using the <odesi> platform [14]. The CCHS is a cross-sectional survey, conducted by Statistics Canada to collect information on health status, health care utilization and determinants of health. The CCHS includes participants 12 years of age and older from all provinces and territories in Canada, but excludes individuals who live on reserves and other Aboriginal settlements, full-time members of the Canadian forces, institutionalized individuals and residents of some remote regions of the country [13].

### Variables

The two outcomes for this study were: 1) having a regular health care provider; and 2) number of medical consultations. Independent variables were selected based on Andersen and Newman’s framework, which conceptualizes utilization of health care based on predisposing, enabling and need factors [15]. The primary exposure was time since immigration. Predisposing factors were sex, age, region of residence, marital status, cultural/racial background and sense of belonging to local community. Sense of belonging to local community was a categorical variable in which respondents answered the question, “How would you describe your sense of belonging to your local community” with four possible responses: very weak, somewhat weak, somewhat strong and very strong. Enabling factors were total household income, education, knowledge of official languages, and having insurance for prescription medications. For the analysis of number of medical consultations, having a regular health care provider was included as an enabling factor co-variate. Need factors were perceived health and number of chronic medical conditions.

### Inclusion and exclusion criteria

Respondents to the CCHS 2015–2016 survey who identified themselves as immigrants and were 18 years of age and older were included in this study. Respondents were excluded if they did not answer the time since immigration question. For the Objective 1 outcome, having a regular health care provider, the sample was further restricted to respondents who answered the regular provider question (“regular provider” sample). For the Objective 2 outcome, number of medical consultations, the sample was restricted to respondents who answered the question regarding number of consultations (“consultations” sample). Cases with missing data on any of the responses were excluded in the data analysis.

### Data analysis and interpretation

The analysis was conducted in Stata software version 16.0 [16]. Sampling weights were applied in the descriptive statistics, bivariate analyses and multivariate analyses, to account for the portion of the population represented by each survey participant. In addition to sampling weights, bootstrap weights were applied for the multivariate analyses to adjust the variance estimates to account for the complex survey design.

Descriptive statistics were reported after applying sampling weights for each sample. In the bivariate analysis for the “regular provider” sample, having a regular health care provider was compared against each independent variable using chi square tests; for the “consultations sample”, number of consultations with medical doctor in the past year was compared against each independent variable using analysis of variance.

For the “regular provider” sample, multivariate logistic regression was used to determine the relationship between having a regular health care provider and all independent variables. For the “consultations” sample, negative binomial regression was used to compare number of consultations with medical doctor in the past year against all independent variables. Interaction terms for time since immigration with age, time since immigration with knowledge of official languages and time since immigration with sense of community, and for sex with age, were also entered into the multivariate model. Only those independent variables and interaction terms found to be significant were included in the final model.

The fit of the models were assessed using Hosmer and Lemeshow goodness of fit test [17, 18], for the “regular provider” sample and chi-square goodness of fit test for the “consultations” sample [19]. Collinearity statistics were determined using the variance inflation factor (VIF) [20] where values above 10 were considered problematic [21].

## Results

### Having a regular health care provider

The “regular provider” sample consisted of 14,077 respondents, representing 88.3% of the total 15,947 immigrants in the CCHS dataset. Respondents were excluded for no response to the outcome (*n* = 39) and for no response to time since immigration (*n* = 1831). Table 1 reports the descriptive and bivariate statistics. Eighty-four percent of respondents had a regular health care provider. All the independent variables examined were found to be significantly associated with the outcome, with the exception of region of residence. Eighty percent of those with a health care provider were established immigrants, relative to 57% of those without a health care provider (Table 1).

**Table 1:** Characteristics of immigrants with and without a regular health care provider (*n* = 14,077)

	n (%)	Has a regular health care provider		***p***-value
		Yes n (%)	No n (%)	
***Independent variables***				
**Primary exposure**				
Time since immigration				< 0.001
< 10 years	3371 (23.9)	2421 (20.4)	950 (42.7)	
≥ 10 years	10,705 (76.1)	9430 (79.6)	1276 (57.3)	
**Predisposing factors**				
Sex				
Male	6883 (48.9)	5568 (47.0)	1315 (59.1)	< 0.001
Female	7184 (51.1)	6283 (53.0)	911 (40.9)	
Age				< 0.001
18–39 years	4443 (31.6)	3216 (27.1)	1226 (55.1)	
40–64 years	6460 (45.9)	5631 (47.5)	828 (37.2)	
65–79 years	2549 (18.1)	2407 (20.3)	142 (6.4)	
≥ 80 years	625 (4.4)	596 (5.0)	29 (1.3)	
Region of residence^a^				0.106
Western Canada	4577 (32.5)	3896 (32.9)	681 (30.6)	
Central Canada	9381 (66.6)	7855 (66.3)	1526 (68.6)	
Atlantic Canada and Northern Territories	119 (0.8)	101 (0.8)	18 (0.8)	
Marital status				< 0.001
Single	2443 (17.4)	1687 (14.3)	756 (34.0)	
Widowed/divorced/separated	1925 (13.7)	1673 (14.2)	251 (11.3)	
Married or common-law	9650 (68.8)	8434 (71.5)	1216 (54.7)	
Cultural/racial background				< 0.001
White	4912 (35.3)	4240 (36.2)	672 (30.5)	
Non-white	9018 (64.7)	7484 (63.8)	1534 (69.5)	
Sense of belonging to local community				< 0.001
Very weak	1006 (7.6)	790 (7.2)	216 (9.9)	
Somewhat weak	3055 (23.1)	2422 (21.9)	632 (29.0)	
Somewhat strong	6390 (48.3)	5432 (49.2)	959 (44.0)	
Very strong	2774 (21.0)	2403 (21.8)	371 (17.0)	
**Enabling factors**				
Total household income				< 0.001
No income or less than $20,000	1036 (7.4)	737 (6.2)	298 (13.4)	
$20,000–$39,999	2363 (16.8)	1947 (16.4)	416 (18.7)	
$40,000–$59,999	2290 (16.3)	1897 (16.0)	393 (17.7)	
$60,000–$79,999	2156 (15.3)	1844 (15.6)	312 (14.0)	
$80,000 or more	6227 (44.3)	5422 (45.8)	805 (36.2)	
Education				< 0.001
Less than secondary school	1342 (9.7)	1215 (10.4)	127 (5.8)	
Secondary school	2747 (19.9)	2311 (19.9)	436 (19.9)	
Post-secondary	9736 (70.4)	8103 (69.7)	1633 (74.3)	
Knowledge of official languages				< 0.001
Not proficient in official languages	590 (4.2)	542 (4.6)	48 (2.2)	
Proficient in official languages	13,479 (95.8)	11,307 (95.4)	2172 (97.8)	
Insurance for prescription medications				< 0.001
No insurance	3886 (27.9)	3194 (27.1)	691 (32.0)	
Insurance	10,045 (72.1)	8573 (72.9)	1472 (68.0)	
**Need factors**				
Perceived health				< 0.001
Poor	474 (3.4)	448 (3.8)	25 (1.1)	
Fair	1189 (8.5)	1067 (9.0)	122 (5.5)	
Good	4458 (31.8)	3855 (32.7)	603 (27.1)	
Very good	4521 (32.2)	3747 (31.8)	773 (34.8)	
Excellent	3385 (24.1)	2684 (22.7)	701 (31.5)	
Number of medical conditions				< 0.001
Zero	5009 (35.6)	3810 (32.1)	1199 (53.9)	
One	3328 (23.6)	2771 (23.4)	558 (25.1)	
Two	1969 (14.0)	1741 (14.7)	228 (10.3)	
Three or more	3770 (26.8)	3530 (29.8)	241 (10.8)	

^a^Western Canada includes British Columbia, Alberta, Saskatchewan and Manitoba, Central Canada includes Ontario and Quebec, Atlantic and North includes remaining provinces and territories

### Multivariate statistics

After controlling for other significant predictors, established immigrants (10 or more years since immigration) were 1.75 times more likely to have a regular health care provider than recent immigrants (less than 10 years since immigration) (OR = 1.75, 95% CI: 1.45–2.10) (Table 2).

**Table 2:** Logistic regression for having a regular health care provider for “regular provider” sample (*n* = 14,077)

	Coefficient (Standard Error)	t statistic	Odds Ratio (95% CI)	***p***-value
**Primary exposure**				
Time since immigration				< 0.001
0–9 years	1		1.00	
≥ 10 years	0.557 (0.095)	5.86	1.75 (1.45, 2.10)	< 0.001
**Predisposing factors**				
Sex				< 0.001
Male				
Female	0.544 (0.082)	6.62	1.72 (1.47, 2.03)	< 0.001
Age				< 0.001
18–39 years				
40–64 years	0.345 (0.100)	3.46	1.41 (1.16, 1.72)	0.001
65–79 years	1.026 (0.158)	6.51	2.79 (2.05, 3.80)	< 0.001
≥ 80 years	1.410 (0.260)	5.43	4.09 (2.46, 6.82)	< 0.001
Marital status				< 0.001
Single				
Widowed/divorced/separated	0.132 (0.162)	0.82	1.14 (0.83, 1.57)	0.414
Married or common-law	0.636 (0.117)	5.42	1.89 (1.50, 2.38)	< 0.001
Sense of belonging to local community				< 0.001
Very weak				
Somewhat weak	0.150 (0.174)	0.86	1.16 (0.82, 1.64)	0.391
Somewhat strong	0.517 (0.165)	3.14	1.68 (1.21, 2.32)	0.002
Very strong	0.584 (0.184)	3.17	1.79 (1.25, 2.57)	0.002
**Enabling factors**				
Total household income				< 0.001
No income or < $20,000				
$20,000–$39,999	0.536 (0.162)	3.30	1.71 (1.24, 2.35)	0.001
$40,000–$59,999	0.725 (01.67)	4.33	2.06 (1.49, 2.87)	< 0.001
$60,000–$79,999	0.946 (0.180)	5.26	2.58 (1.81, 3.67)	< 0.001
$80,000 or more	1.039 (0.156)	6.68	2.83 (2.08, 3.84)	< 0.001
Education				0.033
Less than secondary school			–	–
Secondary school	−0.185 (0.182)	−1.02	0.83 (0.58, 1.19)	0.307
Post-secondary	−0.314 (0.156)	−2.01	0.73 (0.54, 0.99)	0.045
**Need factors**				
Self-perceived health				0.035
Poor		–		
Fair	−0.546 (0.351)	−1.55	0.58 (0.29, 1.15)	0.121
Good	− 0.542 (0.313)	−1.73	0.58 (0.31, 1.08)	0.084
Very good	−0.676 (0.314)	−2.15	0.51 (0.27, 0.94)	0.031
Excellent	−0.734 (0.320)	−2.29	0.48 (0.26, 0.90)	0.022
Number of medical conditions				< 0.001
Zero			–	–
One	0.243 (0.105)	2.31	1.27 (1.04, 1.57)	0.021
Two	0.450 (0.129)	3.49	1.57 (1.22, 2.02)	0.001
Three or more	0.858 (0.142)	6.05	2.36 (1.79, 3.12)	< 0.001

For predisposing variables, being female, older, married/common-law and having a stronger sense of belonging to the community were associated with having a regular health care provider. In terms of enabling factors, after controlling for significant predictors, having higher income and having a post-secondary education were associated with having a regular health care provider. In terms of need factors, after controlling for other predictors, immigrants with very good and excellent self-perceived health were less likely to have a regular health care provider. Conversely, those with a higher number of medical conditions were more likely to have a regular health care provider.

### Number of medical consultations in the past year

The “consultations” sample consisted of 13,912 respondents, representing 87.2% of the total 15,947 immigrants in the CCHS dataset. Respondents were excluded for no response to the outcome (*n* = 223) and for no response to time since immigration (*n* = 1812). The mean number of medical consultations in the past year was 3.37 ± 4.53 (Table 3). The majority of immigrant respondents had been in Canada for 10 or more years (76.0%). All the independent variables examined were found to be significantly associated with the outcome (number of consultations). Recent immigrants had 2.97 ± 4.45 consultations in the past year, compared to 3.50 ± 4.50 consultations among established immigrants.

**Table 3:** Characteristics and mean medical consultations in previous year for immigrants, (*n* = 13,912)

	n (%)	Number of consultations	***p***-value
		Mean (SD)	
**Primary exposure**			
Time since immigration			< 0.001
< 10 years	3340 (24.0)	2.97 (4.45)	
≥ 10 years	10,572 (76.0)	3.50 (4.54)	
**Predisposing factors**			
Sex			< 0.001
Male	6836 (49.1)	2.99 (4.37)	
Female	7077 (50.9)	3.75 (4.64)	
Age			< 0.001
18–39 years	4414 (31.7)	2.76 (4.29)	
40–64 years	6401 (46.0)	3.39 (4.64)	
65–79 years	2497 (17.9)	4.04 (4.38)	
≥ 80 years	600 (4.3)	4.92 (4.92	
Region of residence^a^			< 0.001
Western Canada	4545 (32.7)	3.70 (4.81)	
Central Canada	9249 (66.5)	3.21 (4.37)	
Atlantic Canada and Northern Territories	119 (0.9)	4.19 (4.73)	
Marital status			< 0.001
Single	2430 (17.5)	2.77 (4.36)	
Widowed/divorced/separated	1881 (13.6)	4.02 (4.97)	
Married or common-law	9542 (68.9)	3.41 (4.47)	
Cultural/racial background			< 0.001
White	4831 (35.1)	3.57 (4.60)	
Non-white	8940 (64.9)	3.28 (4.49)	
Sense of belonging to local community			0.029
Very weak	994 (7.6)	3.60 (4.88)	
Somewhat weak	3042 (23.2)	3.33 (4.79)	
Somewhat strong	6329 (48.3)	3.18 (4.16)	
Very strong	2746 (20.9)	3.31 (4.42)	
**Enabling factors**			
Total household income			< 0.001
No income or less than $20,000	1024 (7.4)	3.96 (5.63)	
$20,000–$39,999	2322 (16.7)	3.63 (4.89)	
$40,000–$59,999	2249 (16.2)	3.30 (4.33)	
$60,000–$79,999	2128 (15.3)	3.30 (4.55)	
$80,000 or more	6185 (44.5)	3.24 (4.22)	
Education			< 0.001
Less than secondary school	1305 (9.5)	4.04 (4.72)	
Secondary school	2702 (19.8)	3.50 (4.96)	
Post-secondary	9660 (70.7)	3.24 (4.36)	
Knowledge of official languages			< 0.001
Not proficient in official languages	563 (4.0)	4.15 (4.11)	
Proficient in official languages	13,342 (96.0)	3.34 (4.54)	
Insurance for prescription medications			0.002
No insurance	3880 (27.8)	3.20 (4.52)	
Insurance	9938 (72.2)	3.47 (4.55)	
Has a regular health care provider			< 0.001
No regular health care provider	2221 (16.0)	1.51 (3.38)	
Has a regular health care provider	11,659 (84.0)	3.73 (4.63)	
**Need factors**			
Perceived health			< 0.001
Poor	440 (3.2)	9.24 (8.21)	
Fair	1165 (8.4)	5.74 (6.06)	
Good	4412 (31.8)	3.58 (4.40)	
Very good	4479 (32.3)	2.77 (3.56)	
Excellent	3368 (24.3)	2.30 (3.54)	
Number of medical conditions			< 0.001
Zero	4972 (35.7)	2.02 (3.27)	
One	3314 (23.8)	2.93 (3.81)	
Two	1939 (13.9)	3.61 (4.58)	
Three or more	3687 (26.5)	5.48 (5.63)	

^a^Western Canada includes British Columbia, Alberta, Saskatchewan and Manitoba, Central Canada includes Ontario and Quebec, Atlantic and North includes remaining provinces and territories

### Multivariate statistics

After controlling for all other independent variables, time since immigration was not significantly associated with number of medical consultations (Table 4). In terms of predisposing factors, females age 18–39 had 1.57 times the number of medical consultations as the comparison group (males age 18–39). Relative to immigrants living in western Canada, those living in central Canada had fewer medical consultations. In terms of enabling factors, immigrants with a regular health care provider had more medical consultations, relative to those who lacked a regular health care provider. In terms of need factors, after controlling for other significant predictors, poorer self-perceived health and higher numbers of medical conditions were associated with more frequent medical consultations among immigrant respondents.

**Table 4:** Negative binomial regression for number of medical consultations, for “consultations” sample (*n* = 13,912)

	Coefficient (Standard Error)	t	Incident Rate Ratio (95% CI)	***p***-value
**Predisposing factors**				
Sex and Age				
Male, Age 18–39 years	–	–	–	–
Male, Age 40–64 years	−0.008 (0.083)	−0.10	0.99 (0.84, 1.16)	0.921
Male, Age 65–79 years	−0.035 (0.076)	−0.46	0.96 (0.83, 1.12)	0.642
Male, Age ≥ 80 years	0.164 (0.110)	1.50	1.18 (0.95, 1.46)	0.135
Female, Age 18–39 years	0.451 (0.081)	5.60	1.57 (1.34, 1.84)	< 0.001
Female, Age 40–64 years	0.081 (0.073)	1.11	1.08 (0.94, 1.25)	0.269
Female, Age 65–79 years	−0.055 (0.081)	−0.68	0.95 (0.81, 1.11)	0.495
Female, Age ≥ 80 years	0.002 (0.085)	0.02	1.00 (0.85, 1.18)	0.985
Region of residence^a^				
Western Canada	–	–	–	–
Central Canada	−0.155 (0.037)	−4.12	0.86 (0.80, 0.92)	< 0.001
Atlantic Canada and Northern Territories	0.128 (0.109)	1.19	1.14 (0.92, 1.41)	0.236
**Enabling factors**				
Has a regular health care provider				
No	–	–	–	
Yes	0.732 (0.088)	8.34	2.08 (1.75, 2.47)	< 0.001
**Need factors**				
Self-perceived health				
Poor	–	–	–	–
Fair	−0.396 (0.091)	−4.38	0.67 (0.56, 0.80)	< 0.001
Good	−0.724 (0.071)	−10.24	0.48 (0.42, 0.56)	< 0.001
Very good	−0.847 (0.074)	−11.47	0.43 (0.37, 0.50)	< 0.001
Excellent	−0.912 (0.082)	−11.07	0.40 (0.34, 0.47)	< 0.001

^a^Western Canada includes British Columbia, Alberta, Saskatchewan and Manitoba, Central Canada includes Ontario and Quebec, Atlantic and North includes remaining provinces and territories

## Discussion

Most (84%) immigrant respondents to CCHS 2015–2016 had a regular health care provider. While established immigrants were more likely to have a regular health care provider than recent immigrants, there was no difference in the mean number of medical consultations in the past year of recent and established immigrants. These findings are consistent with the three previous studies that examined the effect of time since immigration on having a regular doctor and found that established immigrants were more likely to have a regular doctor than recent immigrants [5, 8, 9]. Previous secondary data analyses did not examine number of medical consultations in the past year as a measure of health care utilization. While there was no difference in utilization, these findings suggest that the recency of immigration may affect where immigrants seek care and continuity of care. For example, the 2015–2016 CCHS reported that a greater proportion of established immigrants than recent immigrants sought usual care from a doctor’s office (64.4% versus 48.6%) than a walk-in clinic (25.8%% versus 38.3%) [13]. Recent immigrants may also have different care needs than established immigrants. Recent immigrants were significantly younger than established immigrants; 64% of recent immigrants were 18–39 years old compared to 22% of established immigrants. Females age 18–39 had a higher number of medical consultations than the other age groups, likely to due to frequent visits related to reproductive health.

### Shifting immigration policy in Canada

The differences in the profiles of recent and established immigrants in Canada are likely due, in part, to changes in immigration policy over time. There are three main classes of immigrants to Canada: (1) economic class immigrants are selected for their ability to contribute to the nation’s economy, based on a points system; (2) family class immigrants are sponsored by a family member who is either a Canadian citizen or a permanent resident; (3) refugees are accepted into Canada on the basis of a well-founded fear of returning to their country of origin [22]. Over the decades, immigration policy changes in Canada have resulted in the acceptance of more immigrants in the economic class, and fewer from the family class and refugee class [23]. In 2015 and 2016, the years represented in the CCHS dataset used for this study, 57.4% of immigrants who arrived in Canada were in the economic class [24].

This historical perspective of shifting immigration policy is consistent with the findings in this study in terms of the demographics of recent and established immigrants. Recent immigrants were more likely to be younger and to have a post-secondary education than established immigrants (75.8% vs. 68.7%), consistent with the increased emphasis in immigration policies on human capital. Recent immigrants were also more likely to be non-white (80.3% vs. 59.8%), which may reflect the policy and legislative changes in favour of multiculturalism, as well as global economic and political factors.

### Economic and social integration of immigrants

Our findings show that the gap between recent and established immigrants in having a regular health care provider and health care utilization is narrowing [9]. This pattern may stem, in part, from changes to Canadian immigration policy designed to promote the economic and social integration of immigrants [25]. In this study, we found that higher household income was associated with increased odds of having a regular health care provider, and that recent immigrants had lower household income (an indicator of economic integration) than established immigrants. Previous studies have also shown that higher income is associated with higher utilization of health care by immigrants to Canada [5, 6], and fewer unmet health needs [8]. The qualitative literature has also frequently identified economic barriers as an impediment to utilization and access of health care for immigrants to Canada [26–29]. Higher income provides immigrants with resources to help them navigate the Canadian health care system.

Canada is generally regarded as successful in promoting the social integration of its immigrants, based on the national value for cultural diversity held within Canada, and public support for immigration [30] (although immigrants’ actual experience of social integration is varied) [31]. The Multiculturalism Policy and Multiculturalism Act have emphasized the importance of cultural freedom, discouraged discrimination and is perceived as a strategy for the social integration of immigrants [30]. Specific programs are also in place to encourage settlement and integration, such as language training, fast-track citizenship and human rights and equality guarantees [30]. Two variables in the CCHS 2015–2016 dataset are relevant to social integration: sense of belonging to local community and language proficiency. In the present study, immigrants with a higher sense of belonging to the local community were more likely to have a regular health care provider. This may be related to greater supports and resources and greater ability to navigate the health care system for those immigrants who feel more sense of belonging. Adjusting to a new country can be very stressful and have health impacts [32] and consequently, immigrants with more social support [28] and less cultural incompatibility [11] had better utilization. The qualitative literature also points to the importance of culturally appropriate care [26], beliefs by immigrants that the health care system is not adaptable to their beliefs [33], experiences of racial discrimination by health care providers [29], and beliefs by immigrants that providers of a similar cultural background may be able to provide care more appropriate to their needs [34]. These findings suggest that there is a role for interventions both within the health care system and within the larger public policy arena, to promote immigrant patients’ sense of belonging to the local community and utilization of health care, and to ensure that culturally appropriate care is available.

Although knowledge of official languages was not identified as a predictor for either measure of utilization in this study, it is possible that immigrants face language or communication barriers that are more complex than knowledge of official languages and were therefore not captured in this study; for example, even those who report that they are able to conduct a conversation in English or French may have difficulty with the more complex communication required in interactions related to health care. A possible mitigating factor for language barriers in the Canadian context is the availability of same language health care providers. Indeed, among those immigrant respondents to the CCHS 2015–2016 who reported that they had a regular health care provider, 15.6% reported that they communicated with their health care provider in a language other than English or French. Previous qualitative literature reports that immigrants have a preference for same language health care providers [28, 29, 32], even if this meant traveling longer distances [29].

### Limitations

As this was a secondary data analysis, the choice of independent and outcome variables was limited by variables available in the dataset and by the percentage of non-missing responses. Some variables of interest, such as unmet health care needs, rural/urban status and size of community, had a high rate of missing data and therefore could not be included in the analysis. In addition, variables, such as race and time since immigration, had only a limited number of response categories. Although race was captured as a categorical variable in the survey, this information was suppressed in the public data file for confidentiality reasons, and only values of white and non-white were reported. There is a potential for selection bias, because only respondents who answered the questions regarding time since immigration and regular provider were included in the “regular provider” sample, and only respondents who answered the questions regarding time since immigration and number of consultations were included in the “consultations” sample.

The analysis applied sampling weights to account for the portion of the population represented by each survey participant, and bootstrap weights were applied to the multivariate analysis to account for the complex survey design. Although the use of sampling and bootstrap weights were important to yield a result that is representative of the Canadian population, it is important to note that the use of sampling weights entails that the descriptive tables reported do not directly describe the recruited study participants.

Another important limitation is that the CCHS only identifies immigrants based on the question “are you now, or have you ever been, a landed immigrant in Canada”. Respondents may have varying interpretations of what constitutes an immigrant; for example, people who are now Canadian citizens but were originally landed immigrants to Canada. This may lead to some measurement uncertainty. However, the term landed immigrant is a specific immigration status in Canada and as such, will be familiar to most who have gone through the Canadian immigration process. This question also does not distinguish country of origin among immigrants which may be related to the outcomes in this study. In addition, no distinction is made for other classes of immigrants, such as those who were previously refugees but are now landed immigrants. It would be important to study refugees separately from immigrants, since they face specific barriers to utilization of health care related to their refugee status or previous experiences in their home country.

## Conclusions

In this study, data from the 2015–2016 Canadian Community Health Survey were used to examine the relationship between health care utilization and length of time since immigration for immigrants to Canada, using Andersen and Newman’s Framework of Health Service Utilization. After controlling for other independent variables, established immigrants were more likely to have a regular health care provider compared to recent immigrants, but did not differ in terms of the number of medical consultations in the previous year. These findings may stem from shifting immigration policy that have promoted the economic and social integration of immigrants over time, and in turn, facilitated access to health care.

## Data Availability

The datasets analysed during the current study were retrieved from the public use microdata file (PUMF) from the 2015–2016 CCHS, using the <odesi> platform [14], which is available at https://search1.odesi.ca/#/. The dataset is de-identified to ensures the confidentiality of respondents.

## Abbreviations

CCHS: Canadian Community Health Survey
PUMF: Public use microdata file
VIF: Variance inflation factor
